# Knowledge about male circumcision and perception of risk for HIV among youth in Harare, Zimbabwe

**DOI:** 10.4102/sajhivmed.v20i1.855

**Published:** 2019-04-30

**Authors:** Kudzaishe Mangombe, Ishmael Kalule-Sabiti

**Affiliations:** 1 Department of Sociology and Social Anthropology, Faculty of Social Sciences, Great Zimbabwe University, Masvingo, Zimbabwe; 2 Department of Population Studies and Demography, Faculty of Humanities, North-West University, Mmabatho, South Africa

**Keywords:** Knowledge about male circumcision, Perception of risk to HIV, Youth, Zimbabwe, Voluntary medical male circumcision, Voluntary HIV counselling

## Abstract

**Background:**

Male circumcision will require high uptake among previously non-circumcising countries to realise the impact of circumcising in preventing HIV. Little is known about whether youths are knowledgeable about male circumcision and its relationship with HIV prevention and their perception of risk of HIV infection.

**Objective:**

This article aimed to ascertain youth’s knowledge about male circumcision and perception of risk of HIV infection.

**Methods:**

A quantitative study on 784 youth (men aged 15–35 years) was conducted in Harare, Zimbabwe, after obtaining their consent. Multivariate analysis examined the associations between background characteristics and knowledge about male circumcision and the perception of risk of HIV infection.

**Results:**

The results revealed that age was a significant predictor of knowledge about male circumcision among youth in Harare, as was educational attainment and ever having tested for HIV. In addition, youth who had heard of voluntary medical male circumcision were more likely to have high knowledge of male circumcision compared to those who had never heard of it. The results also showed that male circumcision status was associated with higher knowledge about male circumcision compared to those who were not circumcised. The study also found that educational attainment, belonging to the Shona ethnic group, never having tested for HIV and disapproval of voluntary counselling and testing prior to male circumcision were associated with the perception of risk of HIV infection.

**Conclusion:**

The study provides two recommendations: the need to strengthen perceived susceptibility to HIV among the youth and the need for advocacy on the health benefits of male circumcision.

## Introduction

The aim of the study was to identify the sociodemographic factors associated with knowledge about male circumcision and the perception of risk of HIV infection among youth in Harare, Zimbabwe. Male circumcision as a subject is at the helm of HIV prevention. The eastern and southern Africa regions are most affected by HIV, with the number of people on treatment more than doubling since 2010, reaching nearly 10.3 million people, resulting in HIV- and AIDS-related deaths in the region decreasing by 36% since 2010.^1^ According to UNAIDS, the region has also witnessed the largest reduction in new adult HIV infections. There were about 40 000 fewer new adult HIV infections in the region in 2015 than in 2010, a 4% decline.^1^

Although strides have been made concerning improved sexual behaviour in most countries, some countries in sub-Saharan Africa have detected low condom use and an increase in the number of sexual partners in their surveys.^2^ Recent reports show that sexually transmitted infections (STIs) are increasing among certain population groups, including urban youth in Harare.^3^ Considering the higher levels of STIs among the urban youth, there is need to focus attention on knowledge about male circumcision and the perception of risk of HIV infection research in urban areas as a way to curb further HIV and sexually transmitted infections. Both observational and ecological studies have shown that male circumcision reduces female-to-male HIV transmission,^4,5,6^ and three randomised controlled trials^7,8,9^ have shown that male circumcision has the potential to significantly alter the HIV epidemic in countries with high HIV prevalence and low circumcision rates. Thus, in sub-Saharan Africa, there is currently a push for male circumcision to reduce the risk of HIV infection in previously non-circumcising communities.

One of the potential challenges in adopting male circumcision in non-circumcising countries has been a lack of knowledge regarding the benefits in reducing HIV transmission.^10,11,12,13^ Studies on knowledge about male circumcision have been conducted previously,^10,11,14,15^ some of which have found that the perception of risk of HIV infection is a predisposition for willingness to circumcise.^16,17,18,19,20^ However, most of these studies did not exclusively cover male youth age 15–35 years who reside in urban areas.

Little is known about the impact of sociodemographic characteristics on knowledge about male circumcision and perception of risk of HIV infection among youth in Zimbabwe despite the fact that this is a key population in the fight against HIV. This study confines itself to youth aged 15–35 years, which is in line with the African Charter definition.^21^

## Methods

### Design, setting and sample

A cross-sectional study with a closed-ended questionnaire was conducted in Harare among male youth. The sample was calculated using Kish’s^22^ sample size determination. This yielded a target population of 853 men aged 15–35 years. A total of 783 youth were interviewed, yielding a response rate of 92%. The study used the 2012 Zimbabwe Census as the sampling frame, and a three-stage sampling design was employed. Firstly, the process involved the selection of primary sampling units, which were the Enumeration Areas (EAs). The second stage involved the selection of households as secondary sampling units (SSUs). The last stage involved the selection of respondents within the households in the selected EAs. An eligible participant was one male household member between the ages of 15 and 35 years. When multiple households existed within a single dwelling unit, a Kish grid was used to randomly select a responding household, and one member of the selected household was eligible to participate. If the selected household had no eligible respondent, the next household within the same dwelling unit was selected. Thus, a Kish grid was used in the selection of a respondent in a multiple-respondent household and multiple-dwelling units.

### Measures

#### Dependent variables

Knowledge about male circumcision was measured by 10 items measured at the nominal level. The responses for each item were coded 0 and 1, with 0 indicating that an individual did not have knowledge about that particular question and 1 indicating that an individual did have knowledge about it. A knowledge score was obtained by summing up the individual knowledge questions. The score ranged from 0 to 10. The knowledge score was dichotomised. Previous studies have also dichotomised knowledge scores in a similar way.^23^ Studies have confirmed that dichotomisation of continuous variables makes interpretation easy and helps in simplifying analyses or presentation of results.^24,25^ The dichotomisation was done at mean score (7.79). A value of 0–7.78 indicated that the respondents had low knowledge about male circumcision, and a score from 7.79 to 10 indicated that the respondents had high knowledge about male circumcision. For modelling purposes, a dummy variable was created, with 0 indicating respondents had low knowledge about male circumcision and 1 for those respondents who had high knowledge about male circumcision.

Perception of risk of HIV infection was created from responses to a direct question: ‘Do you think you are at risk of HIV infection’? The responses were as follows: ‘Yes, at higher risk’ (assigned a value of 3); ‘Yes, at low risk’ (assigned 2); ‘No, not at risk at all’ (assigned 1).

#### Sociodemographic variables

The respondents were asked about their age in completed years, and these were categorised into 5-year age groups: 15–19, 20–24, 25–29 and 30–35 years. Education was categorised as primary, secondary and higher than secondary. Marital status was categorised as never married, married or living together, and never formerly married. Wealth status was measured using a household goods index, which was recoded into low, medium and high wealth status. The measure was derived from the presence of 10 household assets within the respondent’s household: generator, solar panel, radio, television, refrigerator, non-mobile telephone, computer, washing machine, car and electricity connected to the dwelling unit. Employment status was classified into unemployed or employed. Religion was categorised into mainline Christian, Pentecostal, apostolic sect, other Christian and no religion, while ethnicity was categorised into Shona or other. Respondents were also asked whether they had ever tested for HIV. The responses were ‘yes’ or ‘no’. Respondents were asked whether they approved of voluntary counselling and testing (VCT) prior to circumcision (yes or no). Lastly, respondents were asked whether they had ever heard of voluntary medical male circumcision (VMMC) (yes or no).

#### Data analysis

Data management and statistical analyses were performed using SPSS version 22. The chi-square independence test was used to compare the various sociodemographic and dependent variables. A binary logistic regression model was used to identify predictors of knowledge about male circumcision. Multinomial logistic regression was used to predict the net effect of the predictor variables (background characteristics) on perception of risk to HIV infection. In the model, the reference category for the dependent variable was ‘No, not at risk at all’.

## Ethical consideration

Ethical clearance was granted by the relevant ethics council (Ethics number: North-West University [NWU 00210-14-A9] and Medical Research Council of Zimbabwe [MRCZ/A/1848]). A written informed consent was obtained from all the study participants after describing the objectives of the study to them. In addition, the respondents were assured of confidentially and anonymity.

## Results

### Demographic characteristics of respondents

Table 1 presents the frequency distribution of background characteristics of the respondents. Almost equal proportions were distributed in the four age groups. The highest proportion of the respondents had never been married (63%), had achieved secondary education (78.8%), were employed (56%) and had ever been tested for HIV (65.3%); 90.6% of the respondents were of the Shona ethnic group, and most of them were not circumcised (84.9%). In addition, 33.8% of the respondents were mainline Christians, 78.2% did not approve of HIV testing prior to circumcision, and almost all (96.8%) had ever heard VMMC.

**TABLE 1:** Background characteristics of the study respondents (*n* = 784).

Variable	Frequency	Percentage
**Age group**		
15–19	182	23.2
20–24	229	29.2
25–29	192	24.5
30–35	181	23.1
**Marital status**		
Married or living together	263	33.5
Formerly married	26	3.4
Never married	495	63.1
**Education**		
Primary	37	4.7
Secondary	617	78.8
Higher	129	16.5
**Wealth status**		
Low	276	35.2
Medium	248	31.6
High	260	33.2
**Employment status**		
Employed	439	56.0
Unemployed	345	44.0
**Religion**		
Mainline	265	33.8
Pentecostal	193	24.6
Apostolic sect	169	21.6
Other Christian	83	10.6
No religion	74	9.4
**Ethnic group**		
Shona	710	90.6
Ndebele	23	9.4
**Ever tested for HIV**		
Yes	512	65.3
No	272	34.7
**Approval of VCT prior to MC**		
Yes	171	21.8
No	613	78.2
**Ever heard of VMMC**		
Yes	759	96.8
No	25	3.2
**Circumcision status**		
Yes	118	15.1
No	666	84.9
**Total**	**784**	**100.00**

VCT, voluntary counselling and testing; MC, male circumcision; VMMC, voluntary medical male circumcision.

Table 2 presents the frequency distribution of the respondents’ knowledge about male circumcision. It is evident from the table that more than 9 in 10 respondents (94.1%) knew that it is recommended that a circumcised man still use a condom. Moreover, 7.4% of the respondents knew that male circumcision should be integrated with other HIV prevention methods. About 8 in 10 (81.4%) of the respondents indicated that an HIV-negative woman can contract HIV after having unprotected sex with an HIV-positive uncircumcised man. Reversing the question, about the same percentage (83.7%) indicated that an HIV-negative circumcised man could contract HIV after having unprotected sex with an HIV-positive woman.

**TABLE 2:** Proportion of respondents who answered correctly to the knowledge questions about circumcision (*n* = 784).

Variable	Yes	Percentage
Is male circumcision a surgical removal of the end of the foreskin of the penis?	521	66.5
Is circumcision as good as an ‘invisible condom’ in preventing HIV transmission?	648	82.2
Does male circumcision reduce the chances of transmitting HIV?	668	85.2
Does male circumcision reduce penile cancer?	417	53.2
Are circumcised men still recommended to use condoms?	738	94.1
Does male circumcision improve penile hygiene?	727	7.4
Can male circumcision alone prevent HIV contraction?	726	92.6
Can an HIV-negative woman contract HIV or STI after having unprotected sex with an HIV-positive circumcised man?	638	81.4
Can an HIV-negative circumcised man contract HIV or STI after having unprotected sex with an HIV-positive woman?	656	83.7
After being circumcised must a man abstain from sexual intercourse for six weeks?	367	46.8

A little more than 8 in 10 (82.2%) of the respondents indicated that male circumcision was not as good as an invisible condom. On the other hand, 82.7% indicated that male circumcision reduces the chances of HIV transmission. With regards to male circumcision reducing penile cancer, 53.2% indicated that male circumcision reduces penile cancer. More than 9 in 10 (92.7%) indicated that male circumcision improves penile hygiene, and about the same proportion (92.6%) indicated that male circumcision alone can prevent HIV infection. Fewer than half (46.8%) of the respondents were aware that it was recommended that circumcised men abstain from sexual intercourse for a minimum period of 6 weeks following circumcision. More than two-thirds (66.5%) of the respondents were able to define male circumcision.

Table 3 shows the bivariate relationship between the background characteristics and knowledge about male circumcision. The results showed that there was a significant association between age and knowledge about male circumcision. Knowledge about male circumcision increased with age. A higher percentage of respondents who were aged 30–35 years had high knowledge about male circumcision (88.4%) compared to those who were aged 25–29, 20–24 and 15–19 (80.2%, 79.9% and 71.4%, respectively; *p* < 0.001). A higher proportion of respondents who had attained a higher level of education had high knowledge about male circumcision (93.8%) compared to those who had attained primary and secondary education (77.7% and 70.3%, respectively; *p* < 0.000).

**TABLE 3:** Background characteristics by knowledge about male circumcision.

Variable	Low knowledge (%)	High knowledge (%)	χ-value	*p*	Total
**Age**			**16.325**	**0.001**	
15–19	28.6	71.4	-	-	182
20–24	20.1	79.9	-	-	229
25–29	19.8	80.2	-	-	192
30–35	11.6	88.4	-	-	181
**Marital status**			**10.197**	**0.006**	
Married or living together	13.7	86.3	-	-	263
Formerly married	19.2	80.8	-	-	26
Never married	23.4	76.6	-	-	495
**Education**			**19.618**	**0.000**	
Primary	29.7	70.3	-	-	37
Secondary	22.3	77.7	-	-	618
Higher	6.2	93.8	-	-	129
**Wealth status**			**5.616**	**0.060**	
Low	24.3	75.7	-	-	276
Medium	19.4	80.6	-	-	248
High	16.2	83.8	-	-	260
**Employment status**			**8.184**	**0.004**	
Employed	16.4	83.6	-	-	439
Unemployed	24.6	75.4	-	-	345
**Religion**			**4.425**	**0.352**	
Mainline	17.0	83.0	-	-	265
Apostolic sect	21.9	78.1	-	-	169
Pentecostal	18.7	81.3	-	-	193
Other religion	25.3	74.7	-	-	83
No religion	24.3	75.7	-	-	74
**Ethnic group**			**0.443**	**0.506**	
Shona	23.0	77.0	-	-	710
Other	19.7	80.3	-	-	74
**Ever tested for HIV**			**32.765**	**0.000**	
Yes	14.1	85.9	-	-	759
No	31.3	68.7	-	-	25
**Approve of VCT prior to MC**			**0.072**	**0.788**	
Yes	19.3	80.7	-	-	171
No	20.2	79.8	-	-	613
**Ever heard of VMMC**			**9.268**	**0.002**	
Yes	19.2	80.8	-	-	759
No	44.0	56.0	-	-	25
**Total**	157	627	-	-	784
**Percentage**	20.0	80.0	-	-	100

VCT, voluntary counselling and testing; MC, male circumcision; VMMC, voluntary medical male circumcision.

High knowledge about male circumcision was more common in respondents in the high wealth status group (83.8%) compared to those who belonged to medium and low wealth status (80.6% and 75.7%, respectively; *p* < 0.060). With respect to employment status, 26.6% of respondents who indicated that they were unemployed had low knowledge about male circumcision as compared to 16.4% of respondents who indicated that they were employed.

Eighty-six per cent of the respondents who had ever tested for HIV had high knowledge about male circumcision, compared to 68.8% of those who had never tested for HIV. Finally, 80.8% of the respondents who had heard of VMMC had high knowledge about male circumcision.

### Predictors of knowledge about male circumcision

Table 4 shows the results of the binary regression examining the influence of sociodemographic characteristics and knowledge about male circumcision. The results concerning age and knowledge about male circumcision showed that men aged 25–29 years were significantly less likely to have high knowledge about male circumcision compared to those aged 30–35 years. The odds of young men aged 25–29 years having high knowledge about male circumcision were 42% lower (odds ratio [OR] = 0.576, *p* < 0.1) compared to those aged 30–35 years. Education was significantly related to knowledge about male circumcision. The results showed that the odds of youth with primary education having high knowledge of HIV were 78% lower (OR = 0.223, *p* < 0.05) compared to those with higher education. In addition, youth who reported having secondary education and having high knowledge about male circumcision were 71% lower (OR = 0.292, *p* < 0.05) compared to those with higher education and having high knowledge about male circumcision.

**TABLE 4:** Predictors of knowledge about male circumcision.

Variable	B	SE	Exp(B)
**Age**			
30–35 (R)	-	-	-
15–19	−0.361	0.386	0.697
20–24	−0.316	0.342	0.729
25–29	−0.552	0.321	0.576***
**Marital status**			
Never married (R)			
Married or living together	0.379	0.289	1.461
Formerly married	0.077	0.552	1.080
**Education**			
Higher (R)	-	-	-
Primary	−1.503	0.559	0.223*
Secondary	−1.231	0.402	0.292*
**Wealth**			
High (R)			
Low	−0.209	0.249	0.811
Medium	−0.004	0.248	0.996
**Employment status**			
Unemployed (R)	-	-	-
Employed	0.201	0.231	1.223
**Religion**			
No religion (R)	-	-	-
Mainline	0.426	0.342	1.531
Pentecostal	0.321	0.359	1.378
Apostolic sect	0.245	0.351	1.277
Other Christian	0.024	0.399	1.024
**Ethnic group**			
Other (R)	-	-	-
Shona	0.175	0.316	1.191
**Ever tested for HIV**			
No (R)	-	-	-
Yes	0.660	0.215	1.934*
**Approve of VCT prior to MC**			
Disapprove (R)	-	-	-
Approve	0.187	0.233	1.205
**Ever heard of VMMC**			
No (R)			
Yes	1.255	0.465	3.508*
**Perception of risk to HIV infection**			
No, not at risk at all (R)	-	-	-
Yes, at high risk	−0.085	0.322	0.919
Yes, at low risk	−0.041	0.215	0.960
**Attitude towards male circumcision**			
Unfavourable attitude (R)			
Favourable attitude	0.177	0.197	1.194
**Circumcision status**			
No (R)	-	-	-
Yes	0.577	0.333	1.782***
Constant	0.511	0.810	1.667
Observations	784	-	-
Nagelkerke	0.13.9	-	-
H-L G gof test	0.993	-	-

VCT, voluntary counselling and testing; MC, male circumcision; VMMC, voluntary medical male circumcision; H-L G gof, Hosmer-Lemeshow Goodness of fit test; B, beta values; R, reference category.

* *p* < 0.05,

*** *p* < 0.1

Further, the odds of young men who reported ever having tested for HIV having high knowledge about male circumcision were 93% higher (OR = 1.93, *p* < 0.05) compared to those who had never tested for HIV. With respect to ever having heard of VMMC, the odds of youth who had ever heard about VMMC compared to those who had not having high knowledge about male circumcision were 250% higher (OR = 3.50, *p* < 0.05). Youth who reported being circumcised were more likely (OR = 1.78, *p* < 0.1) to have high knowledge about male circumcision compared to those who were uncircumcised, with 78% higher odds.

Table 5 presents the frequency distribution for respondents’ perception of risk for HIV infection. Fifty-four per cent of the respondents perceived themselves not to be at risk of HIV infection compared to 10.4% who indicated that they were at a higher risk. About a third (35.6%) perceived themselves to be at low risk of HIV infection.

**TABLE 5:** Perception of risk of HIV infection.

Perception of risk to HIV infection	Frequency	Percentage
No risk at all	423	54.0
Low risk	279	35.6
High risk	82	10.4
**Total**	**784**	**100.0**

Table 6 shows that there was a significant association between the background characteristics and perception of risk to HIV infection. Perception of low risk and high risk of HIV infection increased with increase in age. For example, 67.6%, 23.6% and 8.8% of young people aged 15–19 years indicated that they were at no risk, low risk and high risk to HIV infection, respectively. On the other hand, 44.2%, 42.5% and 13.3% of those aged 30–35 years indicated that they were at no risk, low risk and high risk for HIV infection, respectively.

**TABLE 6:** Background characteristics by perception of risk to HIV infection.

Variable	No risk (%)	Low risk (%)	High risk (%)	χ-value	*p*	Total
**Age**				**24.193**	**0.000**	**182**
15–19	67.6	23.6	8.8	-	-	229
20–24	55.5	35.8	8.7	-	-	192
25–29	48.4	40.1	11.5	-	-	181
30–35	44.2	42.5	13.3	-	-	-
**Marital status**				**21.891**	**0.000**	
Never married	58.4	31.9	9.7	-	-	263
Married or living together	47.1	43.0	9.9	-	-	26
Formerly married	23.4	45.8	30.8	-	-	495
**Education**				**17.487**	**0.002**	
Primary	56.8	37.8	5.4	-	-	37
Secondary	57.1	32.5	10.4	-	-	618
Higher	38.0	49.6	12.4	-	-	129
**Wealth status**				**1.970**	**0.741**	
Low	55.1	34.8	10.1			276
Medium	56.0	34.7	9.3	-	-	248
High	50.8	36.8	12.4	-	-	260
**Employment status**				**14.431**	**0.001**	
Employed	48.1	40.8	11.1	-	-	439
Unemployed	61.4	29.0	9.6	-	-	345
**Religion**				**12.154**	**0.144**	
Mainline	53.2	37.0	9.8	-	-	265
Pentecostal	55.4	37.9	6.7	-	-	169
Apostolic sect	58.6	29.6	11.8	-	-	193
Other religion	54.2	31.3	14.5	-	-	83
No religion	41.9	43.2	14.9	-	-	74
**Ethnicity**				**4.964**	**0.084**	
Shona	55.1	34.4	10.5	-	-	710
Other	43.2	47.3	9.5	-	-	74
**Ever tested for HIV**				**30.315**	**0.000**	
Yes	47.3	42.1	10.6	-	-	759
No	43.2	47.3	9.5	-	-	25
**Approve of VCT prior to MC**				**5.103**	**0.078**	
Yes	57.9	31.6	10.5	-	-	171
No	52.9	36.8	10.3	-	-	613
**Ever heard of VMMC**				**3.471**	**0.176**	
Yes	53.4	36.1	10.5	-	-	759
No	72.0	20.0	8.0	-	-	25
Number (Total)	423	279	82	-	-	784
**Percentage**	**54.0**	**35.6**	**10.4**	**-**	**-**	**100**

VCT, voluntary counselling and testing; MC, male circumcision; VMMC, voluntary medical male circumcision.

With respect to marital status, while about a third (30.8%) of formerly married men indicated that they were at high risk of HIV infection, only 10% of never-married men and the same proportion of men married or living together indicated that they were at high risk of HIV infection. Forty-three per cent of married men indicated that they were at a low risk of HIV infection, while 31.9% of never-married men indicated that they were at low risk of HIV infection.

There is a positive relationship between education and risk perception of HIV infection. Nearly half (49.6%) of the respondents with higher levels of education indicated that they were at low risk of HIV infection compared to about a third (32.5%) of respondents with secondary education and close to two-fifths (37.8%) of those with primary education.

There was a significant relationship between employment status and perception of risk of HIV infection (significant at *p* < 0.001). Forty-one per cent of employed youth perceived themselves to be at low risk (40.8%), and 11.2% perceived themselves to be at high risk of HIV infection. On the other hand, 29% of the unemployed youth perceived themselves to be at low risk, compared to 9.6% who perceived themselves to be at high risk of HIV infection.

A similar proportion of respondents who had ever tested for HIV (10.6%) and those who had never tested (9.5%) indicated that they were at a high risk of HIV infection. Additionally, 42.1% of those who had ever tested for HIV reported that they were at a low risk. With those who had never tested for HIV, 47.3% and 9.5% indicated that they were at low risk and at high risk of HIV infection, respectively.

### Predictors of perception of risk of HIV infection

Table 7 shows the results of a multinomial logistic regression model. The results show the relationship between the background characteristics and the perception of risk of HIV infection. In the model, the reference category for the dependent variable is ‘No, not at risk at all’. The results showed that holding other variables constant, the odds of a man perceiving himself to be at a higher risk of HIV infection, relative to perceiving himself to be at no risk of HIV, were higher (OR = 3.13, *p* < 0.05) among formerly married young men than for never-married young men. Youth with secondary education were significantly less likely (OR = 0.535, *p* < 0.1) to perceive themselves to be at higher risk compared to those with higher education. In addition, youth with primary education were significantly less likely (OR = 0.530, *p* < 0.05) to perceive themselves to be at low risk compared to those with higher education.

**TABLE 7:** Predictors of perception of risk of HIV infection.

Variable	Yes, at higher risk 95% CI			Yes, at low risk 95% CI		
	Exp(B)	LB	UB	Exp(B)	LB	UB
**Age**						
30–35 (R)	-	-	-	-	-	-
15–19	0.588	0.223	1.551	0.733	0.379	1.419
20–24	0.561	0.254	1.241	0.886	0.526	1.491
25–29	0.813	0.400	1.651	0.888	0.553	1.426
**Marital status**						
Never married (R)	-	-	-	-	-	-
Married or living together	0.727	0.364	1.452	1.090	0.693	1.714
Formerly married	3.135*	1.054	9.324	1.252	0.455	3.443
**Education**						
Higher (R)	-	-	-	-	-	-
Primary	0.282	0.054	1.467	0.530*	0.223	1.258
Secondary	0.535***	0.264	1.085	0.487	0.306	0.776
**Wealth**						
High (R)	-	-	-	-	-	-
Low	0.905	0.481	1.701	1.123	0.738	1.707
Medium	0.777	0.419	1.441	1.016	0.679	1.522
**Employment status**						
Unemployed (R)	-	-	-	-	-	-
Employed	1.330	0.719	2.461	1.317	0.884	1.963
**Religion**						
No religion (R)	-	-	-	-	-	-
Mainline	0.532	0.225	1.259	0.664	0.366	1.203
Pentecostal	0.343*	0.132	0.891	0.657	0.353	1.222
Apostolic sect	0.598	0.248	1.446	0.518*	0.277	0.970
Other religion	0.719	0.267	1.939	0.582	0.281	1.208
**Ethnicity**						
Other (R)	-	-	-	-	-	-
Shona	0.994	0.406	2.435	0.554*	0.324	0.945
**Ever tested for HIV**						
No (R)	-	-	-	-	-	-
Yes	1.197	0.678	2.113	2.016*	1.377	2.953
**Approval of VCT prior to MC**						
Disapprove (R)	-	-	-	-	-	-
Approve	0.486*	0.238	0.994	1.000	0.683	1.464
**Ever heard of VMMC**						
No (R)	-	-	-	-	-	-
Yes	1.402	0.298	6.590	2.415	0.847	6.890
**Knowledge about MC**						
Low knowledge (R)	-	-	-	-	-	-
High knowledge	1.121	0.601	2.090	1.058	0.696	1.607
**Attitude towards MC**						
Unfavourable attitudes (R)	-	-	-	-	-	-
Favourable attitudes	0.651	0.391	1.085	0.879	0.634	1.218
**Circumcision status**						
No (R)	-	-	-	-	-	-
Yes	0.796	0.399	1.150	0.862	0.343	1.430
Observations	784	-	-	-	-	-
Pearson	0.112	-	-	-	-	-
Deviance	0.833	-	-	-	-	-

VCT, voluntary counselling and testing; MC, male circumcision; VMMC, voluntary medical male circumcision; B, beta values; R, reference category; LB, lower bound; UB, upper bound; CI, confidence interval.

* *p* < 0.05,

*** *p* < 0.1

With regard to religion, the odds of a Pentecostal Christian perceiving himself to be at higher risk were 66% (*p* < 0.05) lower compared to young men who did not profess any religious faith. On the other hand, the odds of apostolic sect youth perceiving themselves to be at low risk of HIV infection were lower by 49% (*p* < 0.05) compared to young men who did not belong to any religion. The odds of Shona youth perceiving themselves to be at low risk of HIV infection compared to young men who belonged to other ethnic groups were 45% lower (*p* < 0.05). Further, the odds of young men perceiving themselves to be at low risk of HIV infection relative to perceiving themselves to be at no risk were 102% higher (OR = 2.01, *p* < 0.05) for those who had ever been tested for HIV than for those who had never been tested for HIV. The odds of youth who approved of VCT prior to male circumcision were less likely (OR = 0.486, *p* < 0.05) to perceive themselves to be at a higher risk of HIV infection compared to their counterparts who did not approve of it.

## Discussion

This study sought to examine youths’ knowledge about male circumcision and perception of risk of HIV infection on the one hand, and selected background characteristics, on the other. In the Zimbabwean context, little is known about the impact of background characteristics on social variables related to knowledge of male circumcision and perception of risk of HIV infection among urban men aged 15–35 years, despite the fact that this is a key subpopulation in the fight against HIV. While some studies have looked at these social variables related to male circumcision,^26^ it is mostly among different subpopulations, only included as predictor variables or not as exhaustive and critically analysed as done in the present study.

Knowledge about male circumcision appeared to increase with age. Youth aged 15–19, 20–24 and 25–29 years all were less likely to have high knowledge about male circumcision. However, only youth aged 25–29 years compared to those aged 30–35 years were less likely to have high knowledge about male circumcision. Studies elsewhere show contrary findings. For example, one study in particular^27^ found no significant association between age and knowledge about male circumcision among young people and adults surveyed in rural Uganda. The differences in these results could have been attributed to the differences in the context of the studies and the definition of youth. ‘Youth’ in the study was defined as men aged 14–24 years and ‘adults’ as those aged 24 years and above.

Furthermore, the findings showed that youth with primary and secondary education were less likely to have knowledge about male circumcision compared to those with a higher level of education. One study observed similar relationships in Zimbabwe among soccer players, with those with higher education levels having more knowledge about male circumcision compared to those with lower levels of education.^28^ Educational attainment predisposes individuals to appreciate health programmes better.^29^

The findings from this study also showed that previously testing for HIV was associated with knowledge about male circumcision. Respondents who had ever tested for HIV were more likely to be knowledgeable about male circumcision. This could be attributed to counselling sessions that happen before and after testing. In Zimbabwe, VCT centres disseminate information about male circumcision. Male circumcision services have also been an integral part of male sexual and reproductive health programmes.^30^ Information about the importance of reduction in the number of sexual partners, provision of condoms, delay in sexual debut and early diagnosis of STIs is disseminated during VCT sessions and in some cases male circumcision.^3^ The likelihood is that those who have ever been tested for HIV were informed or educated about the availability of male circumcision as an option to prevent HIV infection.

In addition, ever having heard of VMMC was found to be statistically significantly associated with knowledge about male circumcision. Youth who had ever heard of VMMC were more likely to have high knowledge of male circumcision compared to those had never heard of it. This could be a result of availability of different mass media advertisement about VMMC. According to a study in Zimbabwe among respondents aged 15–49 years in rural and urban areas, a higher proportion of the respondents had heard of VMMC.^26^

As expected, youth who were circumcised were knowledgeable about male circumcision in comparison to those who were uncircumcised. The present findings are consistent with other research, which found that males who were circumcised were more likely to have high levels of knowledge about male circumcision.^14^ Perhaps the reason why circumcised youth have high knowledge about male circumcision is as a result of intensive information dissemination by health professionals before circumcision takes place in health institutions in Zimbabwe.

The findings suggest marital status had a significant influence on perception of risk of HIV infection. For example, formerly married youths were more likely to perceive themselves to be at higher risk of HIV infection compared to those who had never married. However, previous findings have found that, in general, people tend to underestimate their risk of HIV.^31,32^

In addition, the present study found that education influences perception of risk of HIV infection. For instance, youth with secondary education were less likely to perceive themselves to be at a higher risk of HIV infection compared to respondents with higher education. However, youth with primary education were less likely to perceive themselves to be at low risk of HIV infection compared to those with both secondary and higher education. As education increases, the young men are more likely to perceive themselves to be at high risk of HIV infection, most likely because they understand the dynamics of HIV infection. However, these results are inconsistent with previous studies; for instance, a study among military personnel in Nigeria found an inverse relationship between educational attainment and HIV risk perception.^33^ Results from that study found that those with higher education were less likely to perceive themselves to be at high risk of HIV infection.

Furthermore, the results showed religious variations in perception of risk of HIV infection. Youth who belonged to Pentecostal churches and apostolic sects were less likely to perceive themselves to be at a higher risk of HIV infection compared to respondents with no religion. However, the findings of the current study do not support previous research, which found that these men perceived themselves to be at no risk for HIV infection.^34^ Apostolic sect members usually put stringent restrictions on sexual behaviour, which make them believe that they are not at risk of HIV infection.^34^ For instance, they encourage intermarriage within the church, virginity tests for young girls, polygamy practices and use of the Holy Spirit to detect adultery. This has implications for young men affiliated to the apostolic sect’s perception of HIV infection. Studies have shown that religion impacts on human behavioural and health outcomes.^35^

With regard to Pentecostal Christians, the findings of the current study are consistent with those of a previous study,^35^ which also found that Pentecostal Christians perceived themselves to be at low risk of HIV infection. Plausibly, the association between low perception of risk of HIV infection and Pentecostalism could be attributed to their teachings on nurturing religious experiences and strong synergies encouraged among members. Socialisation of congregants towards more frequent and overlapping interactions can discourage members’ involvement in risky sexual behaviours and hence impact on their perception of risk of HIV infection.^36^

Youth belonging to the Shona ethnic group were less likely to perceive themselves to be at low risk of HIV infection compared to other ethnic groups, which perceived itself to be at no risk of HIV infection. Perhaps the Shona perception of risk of HIV infection is not surprising considering the fact that in Zimbabwe and other sub-Saharan Africa communities they have cultural practices conducive to the spread of HIV such as wife inheritance, which involves relatives of the deceased husband marrying the widow.^37^ In most cases, condoms are not used in this new relationship, because they are not usually used among married couples.^38^ If the widow’s first husband died from HIV, she would be more likely to transmit HIV and/or STIs to the new husband. The practice of wife inheritance is also prevalent in the Bondo District in Kenya.^39^

In addition, those who had ever tested for HIV were more likely to perceive themselves to be at low risk of HIV infection compared to those who had never tested. This relationship could be explained by the fact that HIV is highly stigmatised. Hence, ever taking the test and obtaining negative HIV results makes men aged 15–35 years feel they are not at risk of HIV infection. With respect to approval of VCT prior to male circumcision, respondents who approved of VCT prior to circumcision were less likely to perceive themselves to be at risk of HIV infection compared to those who did not approve of it. Perhaps those who approve of VCT prior to circumcision are youth who regularly check their status and for that reason perceive themselves to be at low risk of HIV infection.

## Conclusion

The findings identified a knowledge deficit about male circumcision among youth with primary and secondary education, indicating the probability of low uptake of male circumcision as an HIV intervention measure in Harare. Furthermore, as education increases, youth are more likely to perceive themselves to be at high risk of HIV infection. The study recommends the need to strengthen perceived susceptibility to HIV across all educational levels, and advocacy is needed on health benefits of male circumcision.
